# Quantitative Morphological Variation in the Developing *Drosophila* Wing

**DOI:** 10.1534/g3.118.200372

**Published:** 2018-06-04

**Authors:** Alexis Matamoro-Vidal, Yunxian Huang, Isaac Salazar-Ciudad, Osamu Shimmi, David Houle

**Affiliations:** * Department of Biological Science, Florida State University, Tallahassee, Florida, United States 32306; † Genomics, Bioinformatics and Evolution Group. Department de Genètica i Microbiologia, Universitat Autònoma de Barcelona, Cerdanyola del Vallès 08193, Spain; ‡ Center of Excellence in Experimental and Computational Developmental Biology. Developmental Biology Program, Institute of Biotechnology, University of Helsinki, PO Box 56, FIN-00014 Helsinki, Finland

**Keywords:** *dachsous*, Geometric Morphometrics, Organ shape, *shifted*, Wing morphogenesis

## Abstract

Quantitative genetic variation in morphology is pervasive in all species and is the basis for the evolution of differences among species. The measurement of morphological form in adults is now beginning to be combined with comparable measurements of form during development. Here we compare the shape of the developing wing to its adult form in a holometabolous insect, *Drosophila melanogaster*. We used protein expression patterns to measure shape in the developing precursors of the final adult wing. Three developmental stages were studied: late larval third instar, post-pupariation and in the adult fly. We studied wild-type animals in addition to mutants of two genes (*shf* and *ds*) that have known effects on adult wing shape and size. Despite experimental noise related to the difficulty of comparing developing structures, we found consistent differences in wing shape and size at each developmental stage between genotypes. Quantitative comparisons of variation arising at different developmental stages with the variation in the final structure enable us to determine when variation arises, and to generate hypotheses about the causes of that variation. In addition we provide linear rules allowing us to link wing morphology in the larva, with wing morphology in the pupa. Our approach provides a framework to analyze quantitative morphological variation in the developing fly wing. This framework should help to characterize the natural variation of the larval and pupal wing shape, and to measure the contribution of the processes occurring during these developmental stages to the natural variation in adult wing morphology.

Those studying the evolution of development have long sought to identify the genes and the cellular processes that give rise to alternative developmental outcomes. Traditionally, the focus of such studies has been on large qualitative differences between species or higher taxonomic entities, or macro evo-devo. However, there is growing interest in complementing such studies with measurement of the quantitative effects of variation at both the genetic and the morphological level within a single species or population (Nunes *et al.*, 2013; Parsons & Albertson 2013). A particularly striking example is Young *et al.*’s (2010) investigation of the relationship between sonic hedgehog (Shh) signaling and quantitative variation in facial shape in the chicken, *Gallus gallus*. Previous work in mammals had generated the hypothesis that disease phenotypes were the result of altered Shh activity. To test this hypothesis, Young *et al.* engineered both high and low Shh expression in the chicken. The estimated dose-response curve confirmed that facial shape in both the embryo and the adult is quite sensitive to Shh variation.

A number of other studies have implicated particular genetic variants as potential causes of quantitative morphological differences using correlative approaches in fish, Lepidoptera, mammals and birds (Mallarino *et al.*, 2012; Nijhout *et al.*, 2014; Salazar-Ciudad & Jernvall, 2010, additional cases reviewed in Parsons & Albertson 2013). To date, such approaches have usually been applied to organisms with direct development, where the identification of homologous structures across developmental stages is straightforward. By contrast, we study a holometabolous insect, *Drosophila melanogaster*, that undergoes a complete metamorphosis between larval and adult structures.

Addressing the question of how changes in development result in quantitative variation of morphology requires quantitative comparisons of the morphology of the developing structures between individuals and between developmental stages (including the adult stage). These comparisons enable us to identify the developmental stage at which morphological variation first appears, and perhaps the developmental mechanism involved.

Flies of the genus *Drosophila* are model examples of a species with a metamorphic event. Like most holometabolous insects, the external structures of the adult are largely formed during larval development, then reshaped during the pupal stage to their final form. In most cases, the precursors of the adult structures, such as legs, eyes or wings are internalized in the larvae, making them difficult to observe. As a result, although the external morphology of *Drosophila melanogaster* is a model for the study of morphogenesis, the relationship between the form of adult organs and the form of their precursors in the larva has rarely been investigated in a quantitative way. One example is Arif *et al.*, (2013), who studied when during development the differences in eye size in relation to face size arose in two Drosophila species; eye size differences arise after the larval stage, but the ratio of eye to face area was established in the larval stage.

In this contribution, we are concerned with the development of the wings of *Drosophila melanogaster*. Critical to this study, the genetics of wing development has been extensively studied, and the processes that influence wing shape determination are, in principle, relatively well known (Matamoro-Vidal *et al.*, 2015). This knowledge suggests that the patterns of protein expression during the larval and pupal phases may mark areas homologous to the structures readily measured in the adult (Blair 2007). Despite this possibility, natural variation in the shape of the wing disc has not been characterized. As a result, it is not known how changes in the larval wing disc shape relate to variation in the adult wing shape, which has been well characterized at the intra and inter-specific levels (Houle *et al.*, 2017).

The fly wing goes through three main developmental stages, represented schematically in Figure 1. First, in the larval stages, the wing tissue is a mono-layered epithelium of cells, the wing imaginal disc, which undergo extensive cell division and tissue patterning. During this period, the number of cells goes from ∼50 to ∼50000, and the major compartments of the wing (ventral, dorsal, anterior, posterior, proximal, distal) are defined. In addition, the tissue is divided into four intervein regions, separated from each other by the provein domains, which are groups of cells expressing a specific set of genes that mark the precursors of the adult wing veins L2 to L5 (Figure 1A). Second, during metamorphosis, the wing imaginal disc is folded such that the dorsal and ventral compartments, which were on the same plane, are now apposed on each other ending up on different planes (Figure 1B). In addition, the tissue expands in the proximo-distal axis giving the tissue a wing-like morphology (Figure 1C). Third, during the late pupal period, the wing hinge contracts while the distal tip is attached to a stiff cuticle, elongating and reshaping the wing along the proximo-distal axis (Figure 1D-E).

**Figure 1:**
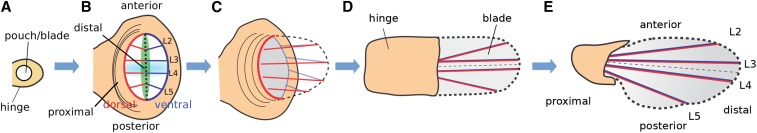
Overview of Drosophila wing development. a. 2^nd^ instar larval disc. b. 3^rd^ instar larval disc with compartments defined by the dorsal/ventral (D/V) and anterior/posterior (A/P) boundaries, provein domains (L2, L3, L4, L5) and morphogen gradients of Dpp, (produced by cells at the A/P boundary – light blue shading) and Wg, (produced by cells at the D/V boundary – green shading). c. Evagination of the disc. The wing pouch folds along its D/V boundary (thick dashed line), apposing dorsal and ventral compartments, and the blade extends and become elongated along the proximal–distal axis. d. Early pupal wing after evagination and expansion. e. Late-pupal wing. The hinge contraction creates tension that drives the elongation of the wing blade. At this stage the shape of the wing blade is similar to adult shape.

Variation in these morphogenetic events must be the source of the natural variation of the adult wing shape but the contribution of each of them is unknown. In this work we provide the first quantitative measurements of the developmental transformation of the late larval wing imaginal disc to the early pupal and adult wing shapes in *Drosophila melanogaster*. We compare shape variation for wing imaginal discs and early pupal wings between wild-type and two mutants in the genes *shifted* (*shf^2^*) and *dachsous* (*ds^1^*/*ds^05142^* ). The developmental effects of the studied alleles are well known (see below), providing an *a priori* expectation of the timing and nature of the effects that these mutations should have on the larval and pupal wing shapes. If we observe shape changes consistent with these expectations, this suggests that measurements of the quantitative effects of genetic variants with unknown developmental roles would help to generate hypotheses about their causal origins.

The *shf* gene codes for a protein involved in the stabilization and diffusion of Hedgehog (Hh) in the larval wing disc. The boundary of Hh signaling in the anterior compartment defines the position of the longitudinal vein L3 along the A-P axis (Blair 2007). In *shf^2^*, Shifted fails to properly stabilize Hh, thus shifting posteriorly the Hh signaling boundary and the position of vein L3. In addition, *shf^2^* wing discs have a reduced expression domain of Dpp, which is a wing growth factor (Glise *et al.*, 2005; Gorfinkiel *et al.*, 2005). We thus expect to observe effects of *shf^2^* on vein patterning and tissue size in the larval wing disc, which is the stage when Hh signaling is active.

*Dachsous* makes an important contribution in orienting cell division during larval development by forming a protein gradient that polarizes the atypical myosin Dachs at the apical cell membrane (Baena-López *et al.*, 2005; Mao *et al.*, 2011). Such polarization orients growth in the direction perpendicular to the dorso-ventral boundary. We thus expect to observe effects of *ds^1^*/*ds^05142^* on larval wing disc shape and size. In addition, the Dachsous protein gradient mediates cell rearrangements and orientation of cell divisions in response to global tissue stress during pupal development in wing and notum epithelia (Bosveld *et al.*, 2012; Sagner *et al.*, 2012). Thus we also expect to observe effects of *ds^1^*/*ds^05142^* on wing shape during pupal development.

## Methods

### Drosophila stocks

**Figure 2:**
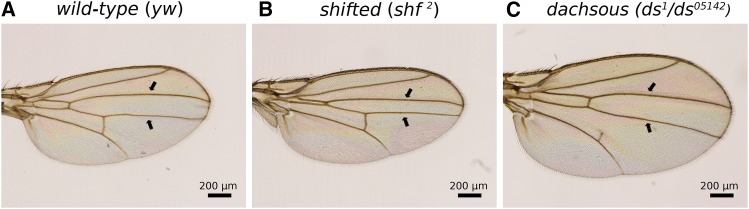
Adult wings for the three genotypes studied. a. *yw*. b. *shf^2^*. c. *ds* (*ds^1^*/*ds^05142^*). Black arrows highlight the longitudinal veins 3 and 4.

The number of wings examined for each condition is given on Table 1. The *yw* flies were used as wild-type. We studied flies homozygous for the *shf^2^* allele (Bloomington # 112), in which the spacing between the third and fourth longitudinal vein is greatly reduced (Figure 2) and a mutant at the *dachsous* (*ds*) gene, which has round wings with increased spacing between third and fourth longitudinal veins (Figure 2). To produce *ds* mutant flies, we generated *trans*-heterozygous individuals for the alleles *ds^1^* (Bloomington # 285) and *ds^05142^* (Bloomington # 11394). Flies homozygous for mutant *ds* alleles have low viability and severe wing overgrowth, making quantitative wing shape measurements challenging. Mutants for study were produced by crossing *ds^1^/CyO*, *P{w[+mC]=Dfd-EYFP}2* and *ds^05142^/CyO*, *P{w[+mC]=Dfd-EYFP}2*. Offspring lacking YFP expression were chosen for measurement.

**Table 1:** Sample size, area means and standard deviations by stage and genotype

Stage	Genotype	Sample size	Area (mm^2^)			
			Anterior	Middle	Posterior	Total
Larva	*yw*	16 (10 ♀, 6 ♂)	0.0053 (0.0008)	0.0016 (0.0003)	0.0029 (0.0005)	0.0100 (0.0014)
	*shf^2^*	8 (5 ♀, 3 ♂)	0.0041 (0.0003)	0.0009 (0.0001)	0.0025 (0.0004)	0.0075 (0.0008)
	*ds*	8 (1 ♀, 7 ♂)	0.0056 (0.0016)	0.0014 (0.0004)	0.0031 (0.0008)	0.0101 (0.0027)
Pupal	*yw*	15 (6 ♀, 9 ♂)	0.0125 (0.0023)	0.0034 (0.0007)	0.0079 (0.0016)	0.0240 (0.0039)
	*shf^2^*	15 (6 ♀, 9 ♂)	0.0107 (0.0030)	0.0014 (0.0004)	0.0055 (0.0015)	0.0177 (0.0047)
	*ds*	12 (4 ♀, 8 ♂)	0.0108 (0.0024)	0.0032 (0.0005)	0.0064 (0.0014)	0.0205 (0.0041)
Adult	*yw*	6 (2 ♀, 4 ♂)	0.375 (0.040)	0.142 (0.017)	0.326 (0.036)	0.843 (0.085)
	*shf^2^*	16 (8 ♀, 8 ♂)	0.383 (0.040)	0.092 (0.011)	0.309 (0.036)	0.784 (0.083)
	*ds*	12 (9 ♀, 3 ♂)	0.486 (0.051)	0.199 (0.019)	0.427 (0.050)	1.112 (0.119)

### Dissections

Larval wing discs were dissected from wandering third instar larvae. The wing discs were fixed with 4% Formaldehyde fixative at room temperature for 20 min, then dissected from the larva.

Pupal wings were dissected 5 h after the white prepupal stage. White prepupae were defined as individuals that had ceased movement, everted anterior spiracles, but had not yet begun tanning of cuticle. Individual white prepupae were picked and reared at 25° until dissection. The pupal wings were fixed with 4% Formaldehyde fixative, left at 4° overnight, and then dissected from the puparia.

Adult wings were dissected from adult flies and mounted with 80% glycerol.

### Immunostaining

We used immunological stains to identify the positions of proveins in larval wing discs and pupal wings. The primary antibodies used were mouse anti-Delta at 1:50 (Developmental Studies Hybridoma Bank, DSHB), and rat anti-Cubitus interruptus at 1:50 (DSHB). The secondary antibodies were as follows: goat anti-mouse IgG-Alexa 568 and goat anti-rat IgG-Alexa 488 were used at 1:200, were (Invitrogen). Immunostaining was performed as described by Matsuda *et al.* (2013).

### Imaging

The fluorescent images were obtained with a Zeiss LSM700 confocal microscope. Adult wing images were obtained with a Nikon Eclipse 90i. Scale information was recorded in the image.

### Landmarks and semi-landmarks

**Figure 3:**
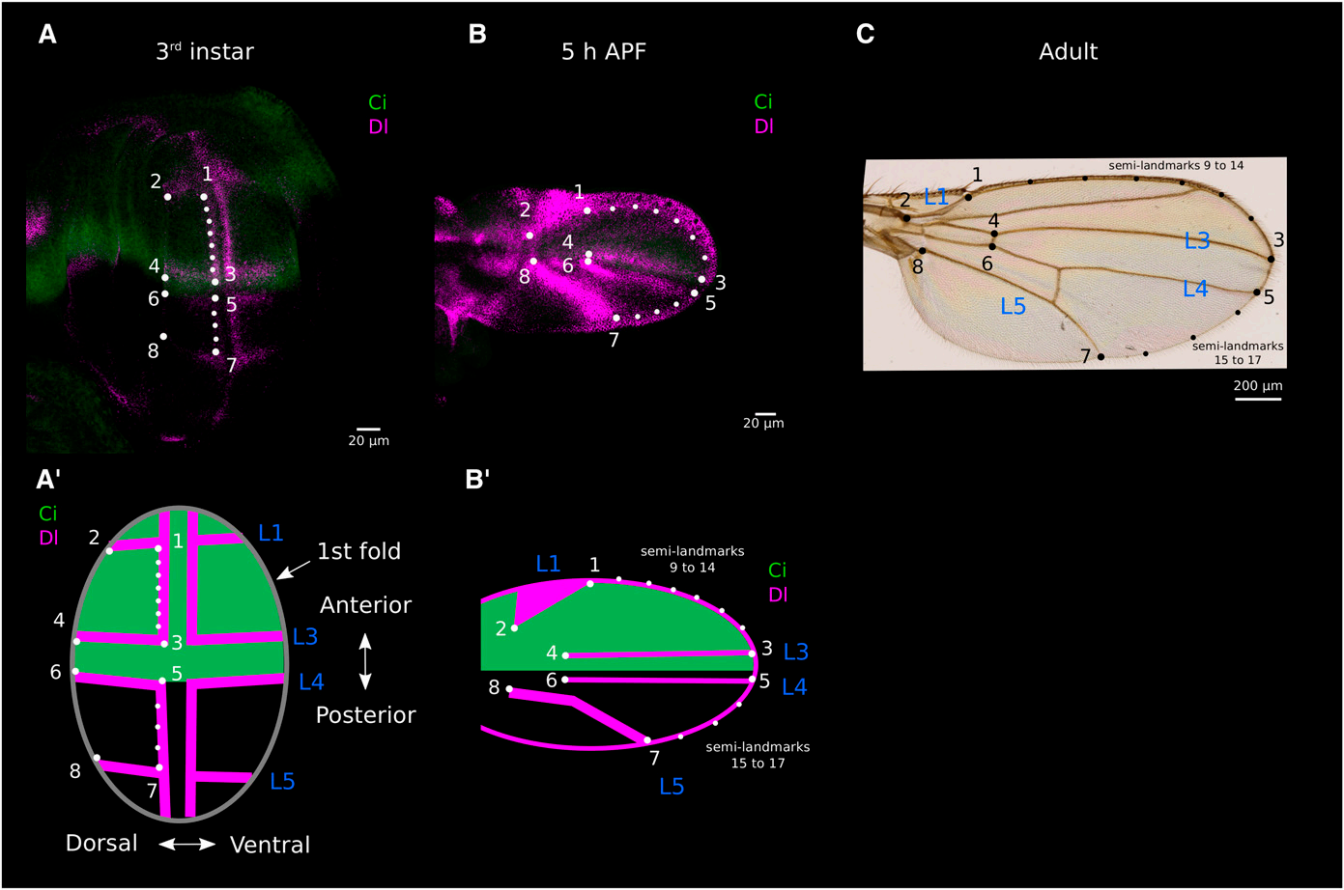
Landmarks and semi-landmarks used for the morphometric analyses. a. 3^rd^ instar larval wing stained with antibodies against Cubitus interruptus (Ci, green) and against Delta (Dl, magenta). Wing shape was measured by gathering 8 landmarks (big white dots numbered 1-8) and 9 semi-landmarks (smaller white dots). a’. Diagram of a 3^rd^ instar larval wing showing how the Delta staining (proveins and D/V boundary) and the 1^st^ fold were used for landmarks and semi-landmarks positioning. b. Pupal wing at 5 h after puparium formation (APF) with same staining than in ’a’ and landmarks/semi-landmarks positions hypothesized to be homologous to those in ’a’. b’. Diagram of 5 h APF pupal wing showing how Delta staining (proveins), and the wing margin were used for landmarks and semi-landmarks positioning. c. Dorsal adult wing with landmarks and semi landmarks positions hypothesized to be the same than in ’a’ and ’b’.

Size and shape of 3^rd^ instar wing discs, 5 h pupal wings, and adult wings were characterized by a set of 8 landmarks and 9 semi-landmarks on each specimen (Figure 3), using *tpsUtil* and *tpsDig2* software (http://life.bio.sunysb.edu/morph) for the discs and pupal wings; and using *Wings4* (Houle *et al.*, 2003; http://bio.fsu.edu/∼dhoule/wings.html) for the adult wings.

The positions of the landmarks were defined using molecular and morphological markers (Figure 3). For the former, we used immunostaining showing the Cubitus interruptus (Ci) and Delta (Dl) territories in wing discs and 5 h pupal wings. The gene *ci* is expressed in the anterior wing whereas *dl* is expressed in two stripes of cells following the dorso-ventral boundary, as well as in the provein precursors of veins 1, 3, 4 and 5 (Biehs *et al.* 1998; Cook *et al.* 2004). The morphological markers were the first fold of the wing pouch, the margins of the pupal and adult wings, and the veins of the adult wings.

For the wing discs, four landmarks (1, 3, 5 and 7) were placed in the distal part of the tissue, at the intersections of the DV boundary with the proveins L1, L3, L4 and L5, respectively. Four other landmarks (2, 4, 6 and 8) were placed at the distal tips of the proveins 1, 3, 4 and 5, respectively, which coincide with the intersections of these proveins and the first fold of the pouch. Note that the position of vein L4 coincides with the end of the anterior compartment (shown by Ci expression). In addition, two sets of semi-landmarks were placed on the DV boundary. The first set (9-14) was placed along the portion of the DV boundary contained within proveins L1 and L3, and the second (15-17) was placed along the curve connecting L4 and L5. Data were initially collected for ventral and dorsal compartments of the wing disc. However, the ventral compartment was found to be quite variable because this part of the disc starts to evert very early. Thus only the data for the dorsal disc were considered.

For the pupal wings, four landmarks (1, 3, 5 and 7) were placed at the intersections of proveins L1, L3, L4 and L5 with the wing margin, and four others (2, 4, 6 and 8) at the proximal tips of proveins L1, L3, L4 and L5. As in the larval wing discs, two sets of semi-landmarks were placed along the wing margin between L1 and L3 and between L4 and L5.

For the adult wings, four landmarks (1, 3, 5 and 7) were placed at the intersections of veins L1, L3, L4 and L5 with the wing margin. Landmarks 4 and 6 were placed at the intersections between the anterior cross-vein and veins L3-L4; landmark 8 was placed at the intersection between veins L5 and L6 (anal crossvein) and landmark 2 at the proximal end of vein L1. Again, two sets of semi-landmarks were placed along the wing margin between L1 and L3 and between L4 and L5.

### Shape analysis

The combined data on landmark and semi-landmark positions from the larval discs and the pupal and adult wings was subjected to generalized Procrustes superimposition (Rohlf & Slice 1990), using the program tpsRelw (http://life.bio.sunysb.edu/morph/index.html). Procrustes superimposition scales forms to the same size, translates their centroids to the same location, and rotates them to minimize the squared deviations around each point. This separates the useful size and shape information from the nuisance parameters introduced by the arbitrary location and rotation of the specimens within the images. The positions of the semi-landmarks were slid along each dorsal-ventral boundary segment defined by the boundary landmarks to minimize deviation along the segment using the standard model in tpsRelw. After registration and sliding, the resulting shape data have 18 degrees of freedom.

Analysis of shapes using tpsSmall (http://life.bio.sunysb.edu/morph/index.html) shows that Euclidean distances were extremely highly correlated with Procrustes distances (r = 0.999964), despite the wide differences in shapes of larval, pupal and adult forms. We performed a principal component analysis on the shape data, retaining 18 PC axes for further analyses.

Traditional outlier detection using distance from the mean is challenging in multivariate space, as undetected outliers will alter the mean and inflate the covariance structure that is used to calculate the distance of each observation from the mean. We diagnosed the presence of outliers using Minimum Covariance Determinant (MCD) approach (Rousseeuw & Van Driessen 1999) which uses a random subset of data that minimizes estimated covariance. This procedure was implemented using the Diagnostics option in the Robustreg procedure in SAS employing a dummy dependent variable (Wicklin 2012).

We performed outlier detection for the first 5 shape principal component axes within each genotype and stage. Specimens more than 3 S.D.s away from the robust means (*i.e.*, estimated with MCD approach) were identified as outliers. Images of putative outliers were re-examined to determine the source of the unusual measurements. For adult wings, wings with relatively extreme *ds* and *shf^2^* phenotypes were identified as outliers. We retained these in the data, as the deviations were relatively modest. For larval wings, one *shf^2^* outlier appeared to have a damaged disc, and was omitted. Four pupal outliers (two *ds*, and two *yw*) greater than 6 S.D. from the robust mean were omitted because of unusual staining patterns, or distortions of the epithelia. The final shape data set consists of 108 specimens. No univariate outliers for size (area or centroid size) were detected using Grubb’s test.

To test whether genotypes differed in the developmental transformations they undergo from larval to pupal to adult form, we used a multivariate analysis of variance (MANOVA). Type III sums of squares and cross-products were used to calculate test statistics. The variance of shape was very different among developmental stages, which violates the assumption of homogeneous variances used for conventional statistical tests. To provide an alternative test, we performed MANOVAs of data randomized to make the null hypothesis of no effect true. We first decomposed each observation into the grand mean, plus residuals corresponding to stage, genotype, and genotype by stage data, and residual as follows

$$S_{sgki}={\bar{S}}_{k}+{\bar{R}}_{sk}+{\bar{R}}_{gk}+{\bar{R}}_{sgk}+\varepsilon_{sgki}$$

where *s* indexes developmental stage, *g* indexes genotype, *k* indexes the shape variable, i the individual, the overbar indicates a mean shape, and $\varepsilon_{sgki}$ is the deviation of the individual from the stage-genotype mean. We then randomized just the deviations used to test a particular hypothesis, holding all other aspects of the observation constant. For example, to test for stage by genotype interactions, we randomized ${\bar{R}}_{sgk}$ values among individuals within stages. We retained values of Wilk’s lambda, the standard test statistic used for MANOVA, from 1,000 randomized analyses. To test a hypothesis, quantiles of the randomized Wilk’s lambda distribution were compared with the Wilk’s lambda obtained from the analysis of the observed data.

### Scalar measures

The standardized distances between the 28 possible pairwise combinations of the 8 landmarks were obtained from the Cartesian coordinates of the landmarks standardized by the centroid size. Centroid size is proportional to the square root of wing area. In addition, we measured the standardized lengths for a portion of the anterior margin using landmarks 1-3 and semi-landmarks 9-14, and for a portion of the posterior margin using landmarks 5-7 and semi-landmarks 15-17 (Figure S1).

Three areas were calculated using the surveyor’s formula for calculating areas of polygons. The first area was obtained by calculating the area of the regular polygon within the landmarks 1-4 and semi-landmarks 9-14, thus obtaining a proxy of the anterior wing area (’Anterior’). The second areas is for the polygon defined by the landmarks 3-6 which contains the region within the longitudinal veins L3 and L4 (’Middle’). The third area is the one of the regular polygon defined by landmarks 5-8 and semi-landmarks 15-17, and gives a proxy of the posterior wing area (’Posterior’) (Figure S1).

Standardized lengths and areas were compared between developmental stages and between genotypes by calculating means ratios. Values of variance for these ratios were obtained by bootstrapping the data. For example, change in the standardized length between landmarks 2 and 8 (stlen28) during the larval to pupal transition in the *yw* genotype was calculated with the following procedure: individual values for stlen28 in the *yw* pupal wings population were re-sampled with replacement a number of times equal to the number of individuals in the population. The mean on the re-sampled data were calculated and divided by the mean obtained by the same approach on the *yw* larval wing population. This procedure was repeated 1000 times providing thus a distribution of values for the ratio of stlen28 (pupa) / stlen28(larva) of the *yw* genotype. Scalar measures were computed and plotted using R version 3.3.0 (R Core Team 2016).

### Analyses

Statistical tests were carried out in the GLM procedure in SAS (SAS Institute 2011), assuming that stage, genotype and sex are fixed factors. Type III sums of squares and cross-products were used for statistical testing. When interaction terms had *P* > 0.2, they were dropped from the final model. Post-hoc comparisons among genotypes were adjusted within traits for multiple comparisons using the Tukey-Kramer method. The standard errors of ratios of wing areas were approximated using standard formulas for the variance of a ratio, and tests for differences among ratios assumed that the differences are normally distributed. To do this, we had to assume that the covariance of areas between stages is 0, leading to an overestimate of the variance, and conservative tests for differences among the ratios.

### Data Availability

File S1 (available on the FigShare depository) contains the landmark data used for the analyses. Supplemental material available at Figshare: https://doi.org/10.25387/g3.6379235.

## Results

### Size changes

Means and standard errors for areas are shown in Table 1. Analysis of log10 area in a model with stage, sex and genotype as factors shows that stage (*P* < 0.0001), genotype (*P* < 0.0001) and sex (*P* = 0.0006) are all highly significantly different from 0. In addition, there is a modestly significant interaction between genotype and sex (*P* = 0.034), and a marginally significant interaction between stage and genotype (*P* = 0.07).

### Stage to stage wing area increases markedly but in a different way Between genotypes

The dorsal area expands markedly during development (File S2). The ratios (± SE) of pupal to larval wing areas are similar for the three genotypes, increasing by factors of 2.0 ± 0.2 (*ds*), 2.3 ± 0.2 (*shf^2^*) and 2.4 ± 0.1 for the wild-type (*yw*). At the pupal to adult transition the increases in wing area are all different from each other, increasing by factors of 54 ± 4 for *ds*, 44 ± 3 for *shf^2^*, and 35 ± 2 for *yw*. Wing area increases between the larval and adult stages by factors of 109 ± 11 (*ds*), 103 ± 5 (*shf^2^*) and 84 ± 5 (*yw*).

### Shape changes

Randomized MANOVA analysis showed a highly significant effect of genotype over stages, as our test statistic was much less than all 1,000 randomized data sets without a genotype effect (Wilks’ λ=0.068, minimum randomized Wilks’ λ=0.230). This result demonstrates that some of the shape differences among genotypes are consistent across all three stages. However, there was also a highly significant stage by genotype interaction (Wilks’ λ=0.074, minimum of 1,000 randomized Wilks’ λ=0.609), which demonstrates that there are changes in the relationships among genotypes over stages. Thus, we first analyze the changes in shape over stages that are shared by all genotypes and then shape changes specific to each genotype.

### Changes in shape Over stages that are shared by all genotypes

#### Developmental trajectories are similar among genotypes:

To assess the similarities in the direction of shape change between genotypes among stages, we calculated the angles between shape change vectors. To do this, we calculated the average direction of shape change between stages for each genotype as the difference in mean phenotype across each transition. We then calculated the angles between these shape change vectors, with the results shown in Table 2. Completely independent shape changes would have an angle of 90, while identical transformations have an angle of 0. The angles are quite close to the minimum of 0, and suggest that genotypes are undergoing similar transformations. In particular, the transformations from larval to adult shapes differ on average by just 8 degrees. Angles involving pupal shapes are generally larger, which probably reflects the larger variation in pupal shape than the other two stages, with correspondingly larger uncertainty as to the true pupal mean.

**Table 2:** Angle in degrees between the vectors of shape changes for each genotype

Comparison	*yw vs. ds*	*yw vs. shf^2^*	*ds vs. shf^2^*
larval to pupal	9.9	16.0	16.8
pupal to adult	25.4	14.3	19.4
larval to adult	7.8	6.3	9.8

#### Larval, pupal and adult shapes are distinct, With major shape changes occurring During wing eversion:

**Figure 4:**
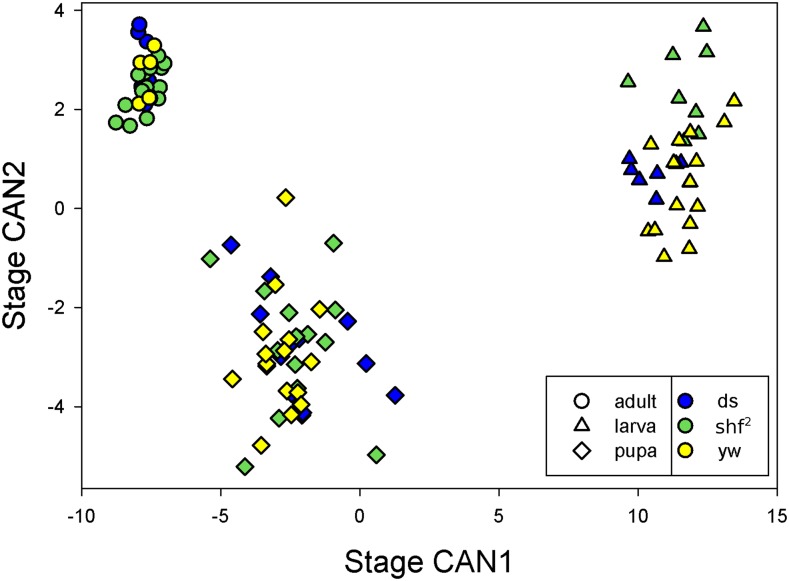
Scores for shape on canonical axes chosen to discriminate stages.

To examine the relative shapes of individuals at each stage, we performed canonical discriminant analyses on the principal components of the shape data. Figure 4 plots the scores on the first and second canonical axes when the discriminant analysis used developmental stage as the classification variable. Larval, pupal and adult shapes are distinct. Note that the within stage individuals variability is quite different for the larval, pupal and adult stages. As a result, standard statistical tests across stages are likely to be biased. A MANOVA on the shape data showed that the effect of stage was highly significant (Wilks’ λ=0.00159, num df = 38, den df = 158, *P* < 0.0001).

To get a sense for the size of stage effects, we calculated the matrix of Euclidean distances in shape space (centroid size units) among individuals in each stage/genotype combination, with the results shown in Table 3. The mean distance between individuals within stages is 0.13 (0.14 within larvae, 0.19 with pupae, 0.07 within adults,), while it is 0.51 between larval and pupal shapes, 0.32 between pupal and adult shapes, and 0.74 between larval and adult shapes. Thus, pupal shape is more similar to adult shape than to larval shape, suggesting that eversion and folding has a larger effect on shape than pupal development.

**Table 3:** Mean shape distance between individuals in each stage/genotype combination. Values are the mean Euclidean distances between the 34 element vector of shape coordinates. Diagonals are the average distances between different individuals of the same genotype and stage

		Larva			Pupa			Adult		
		*yw*	*ds*	*shf^2^*	*yw*	*ds*	*shf^2^*	*yw*	*ds*	*shf^2^*
**Larva**	*yw*	0.12	0.14	0.16	0.56	0.50	0.50	0.77	0.75	0.77
	*ds*		0.10	0.20	0.54	0.47	0.48	0.75	0.73	0.76
	*shf^2^*			0.10	0.54	0.50	0.48	0.73	0.72	0.73
**Pupa**	*yw*				0.15	0.21	0.21	0.28	0.24	0.30
	*ds*					0.17	0.22	0.36	0.31	0.38
	*shf^2^*						0.18	0.34	0.32	0.35
**Adult**	*yw*							0.02	0.11	0.07
	*ds*								0.04	0.16
	*shf^2^*									0.02

#### Stage to stage wing shape variation is characterized by a continued lengthening of the distal wing along the proximo-distal axis and a shortening of the proximal wing along the anterior-posterior axis:

**Figure 5:**
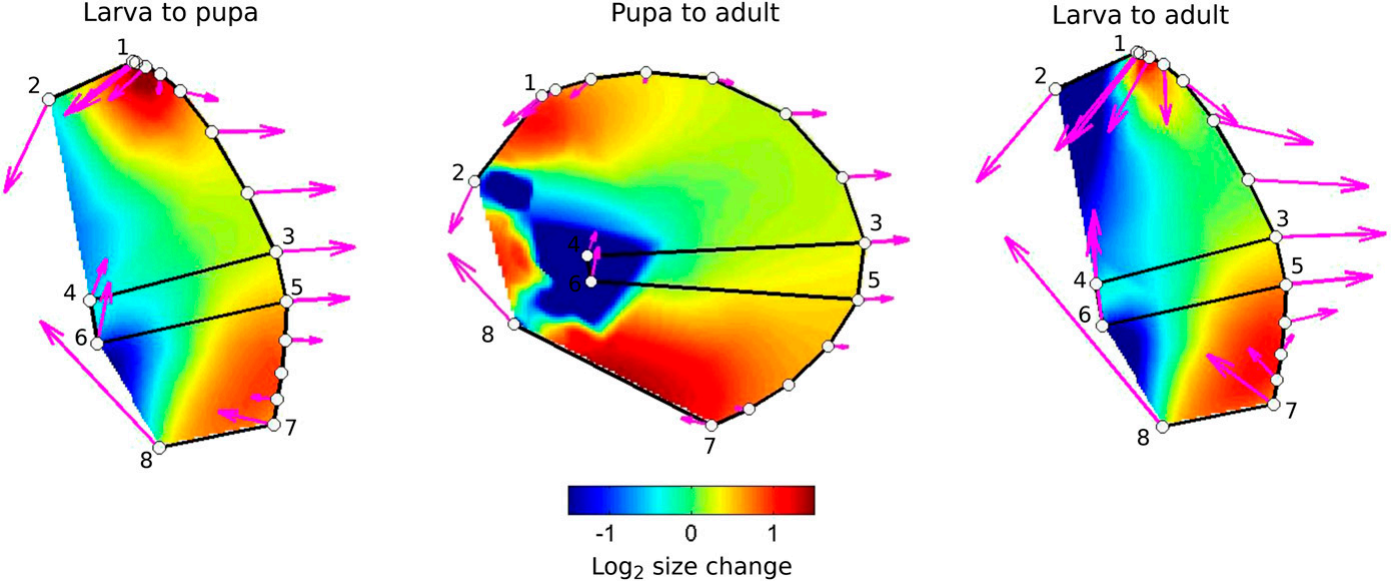
Differences among stages. Colors represent inferred changes in the relative areas of parts of wing necessary to transform the form from the earlier stage (*e.g.*, larva) to the later (*e.g.*, adult) stage. Expansions and contractions are shown on a log_2_ scale, the orange at +1 represents a doubling to relative area, while blue at -1 represents a local halving of area. Magenta arrows represent the pattern of change in location of landmarks (numbered 1 to 8) and of semi-landmarks.

To enable visualization of shape differences, we used the program Lory (Márquez *et al.*, 2012) to show one pattern of relative expansion or contraction that can transform one mean shape into another. Figure 5 plots stage transformations. The magenta arrows represent changes in relative locations of landmarks, while the colors between landmarks represent the inferred expansion and contractions that can bring about the changes in landmark positions. It is important to realize that these represent only shape change, and not size change. The transformation shown is a hypothesis, as other patterns of expansion and contraction can lead to the same shape change at the measured locations.

The overall pattern of shape change is that the distal parts of the wing, closest to veins L3 and L4, move to the right in the figure, shown by the magenta arrows, while the proximal anterior and posterior parts of the boundary are drawn together and to the left, relative to the rest of the wing. File S3 shows the same data in a movie as a transformation of the outline of the wing between stages.

Our linear measurements show that during the larval to pupal phase, shape change is characterized by a narrowing of the tissue along the anterior-posterior axis (*e.g.*, reduction in the relative distances between pairs of landmarks 2-8; 1-8; 4-8 – Figure S2A) and by an expansion in the direction of the proximal – distal axis, as illustrated by the increase in the relative distances between the pairs of landmarks 7-8; 5-8; 3-8, and by the lengthening of the anterior and posterior margins (Figure S2A). This pattern of shape change is continued into late pupal development, with a pronounced constriction along the anterior-posterior axis in the proximal parts of the wing (∼50% decrease in the distance between the pairs of landmarks 2-8;1-4;1-8 and 1-6) and elongation along the proximal-distal axis (Figure S2B).

### Changes in shape specific to each genotype

#### Wing shape is different Between genotypes at all stages:

**Figure 6:**
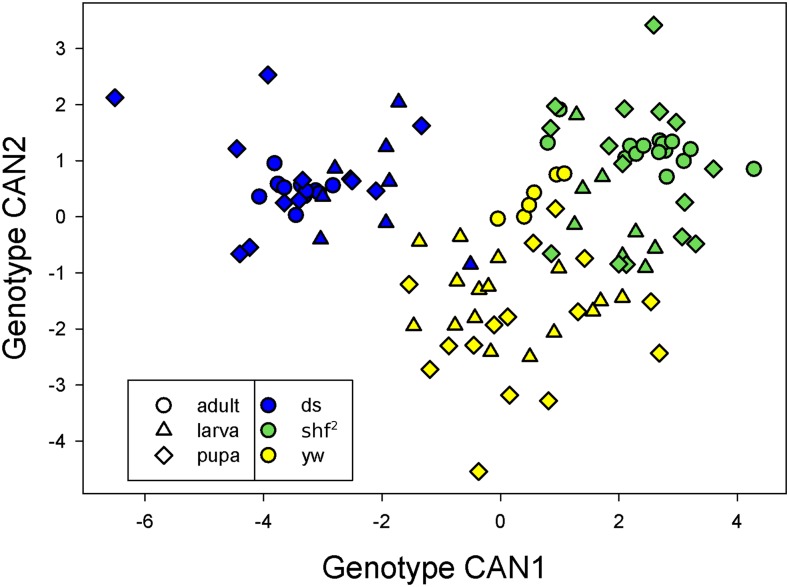
Scores for shape on canonical axes chosen to discriminate genotypes.

Figure 6 shows the scores on the first and second canonical axes when the discriminant analysis used genotype as the classification variable. Genotypes are well separated on these axes, with a few exceptions. We tested for differences in shape between genotypes within stages using a multivariate analysis of variance, with the results shown in Table 4. In all three stages, there were highly significant differences among genotypes.

**Table 4:** Results from MANOVA of shape data within each stage

Stage	Effect	Num df	den df	Wilks’ λ	P
Larva	Genotype	36	18	0.004	<0.0001
	sex	18	9	0.261	0.30
	Genotype by sex	36	18	0.108	0.50
Pupa	Genotype	36	38	0.030	<0.0001
	sex	18	19	0.417	0.20
	Genotype by sex	36	38	0.177	0.13
Adult	Genotype	36	22	0.0002	<0.0001
	sex	18	11	0.207	0.08
	Genotype by sex	36	22	0.082	0.15

The sizes of genotypic effects on shape obtained from the matrix of Euclidean distances in shape space (Table 3) show that the differences in shape among genotypes within stages are less dramatic than the between stage differences. For larvae the average distance between different individuals with the same genotype is 0.11, while the differences among individuals of different genotypes is 0.17. In pupae the within genotype distances average 0.17, while the among genotype distances average 0.22. Adults of the same genotype average just 0.03 in distance, while the among genotype distances average 0.11. This is likely to be due to higher accuracy of measurements in adults.

#### shf mutation affects wing shape early in development whereas ds mutation affects shape Throughout development:

**Figure 7:**
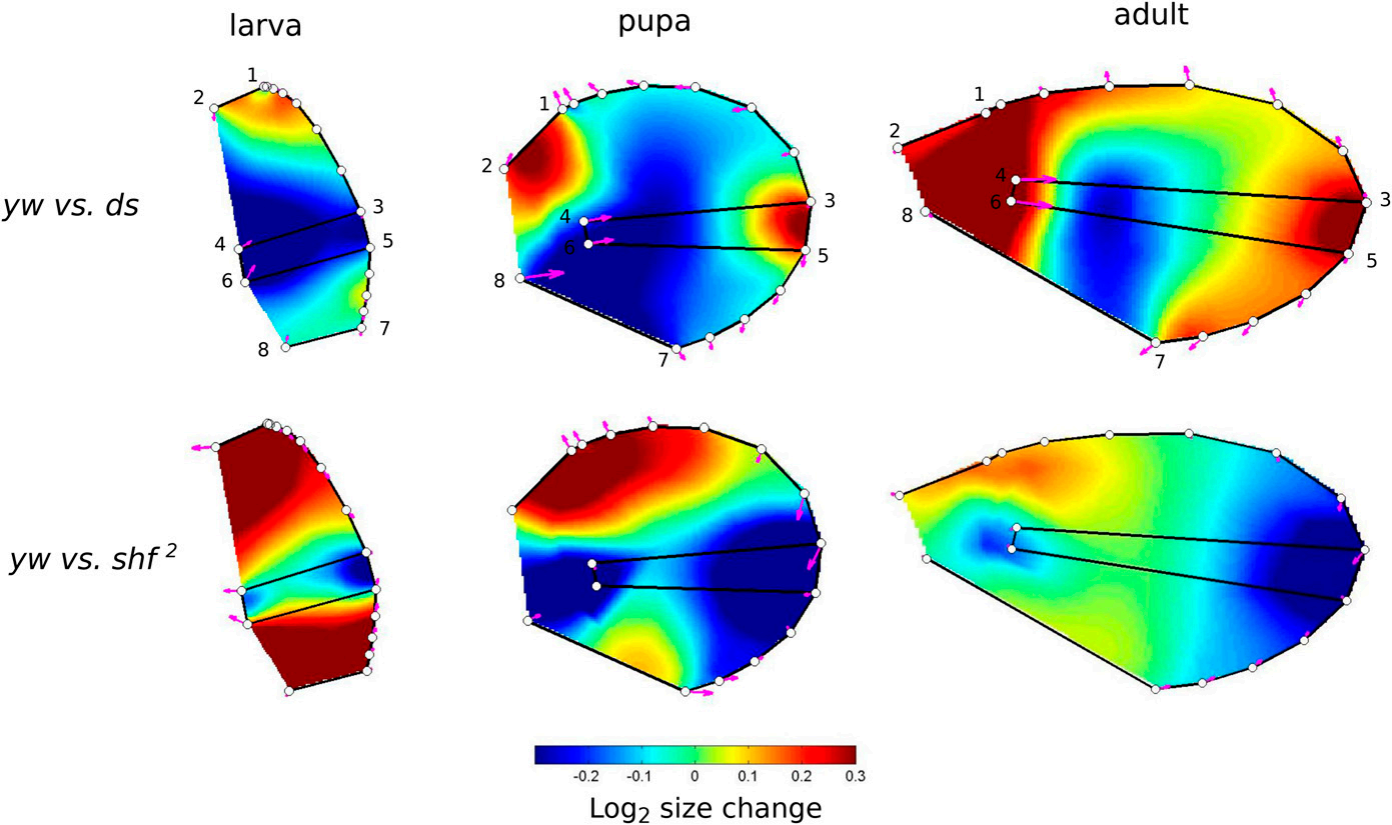
Differences among genotypes within stages. The *yw* genotype is taken as the reference, and colors represent changes in relative area necessary to transform the wing at a given stage (larva, pupa or adult) into the other two genotypes. Note that the scale differs from that in Figure 5. The top of the scale represents an increase by a factor of 1.23.

Figure 7 plots the differences between genotypes relative to the *yw* genotype. We used *yw* as the reference as the mutations it carries are not known to affect wing development. Comparison of *yw* and *ds* suggest that differences in the anterior- and posterior-most regions that will become proximal in the adult exist from the larval stage, but that the majority of the difference between these genotypes arise during pupal development, and the peripheral areas of the blade expand more in *ds* mutants than *yw*. Comparison of *yw* and *shf^2^* suggests that the region between L3 and L4 is markedly smaller in *shf^2^* from the larval stage. This contraction persists, but is balanced principally by an expansion of the proximal part of the wing anterior to L3 in later stages.

To diagnose when these differences arise we examined the ratios *ds*/*yw* and *shf^2^*/*yw* of the standardized lengths and areas. These ratios were first calculated on the adult data to see what is different in adult wings between *yw* and the mutants, and then on the larval and pupal wing data to check when the variation observed in the adults appears during development. The ratios *ds*/*yw* of the standardized lengths for the adult wings are shown in Figure S3A. The *ds* adult wings are narrower relative to *yw* along the P/D axis in the distal part, as well as broader along this same axis in the proximal part. This is shown by the shift of landmarks 4 and 6 toward the distal parts. These two landmarks indeed have higher relative distances with respect to landmarks 1, 2 and 8, as well as lower relative distances with respect to landmarks 3, 5 and 7. In addition, *ds* wings are broader along the anterior-posterior axis, as shown by increase in relative distances between the pairs of landmarks 4-6 and 3-5. Regarding the areas (Figure S3B), our data show that *ds* wings are 1.3 times bigger than *yw*, and this is due to an increase in all the three areas measured with a slightly more important contribution of the “Middle” area.

**Figure 8:**
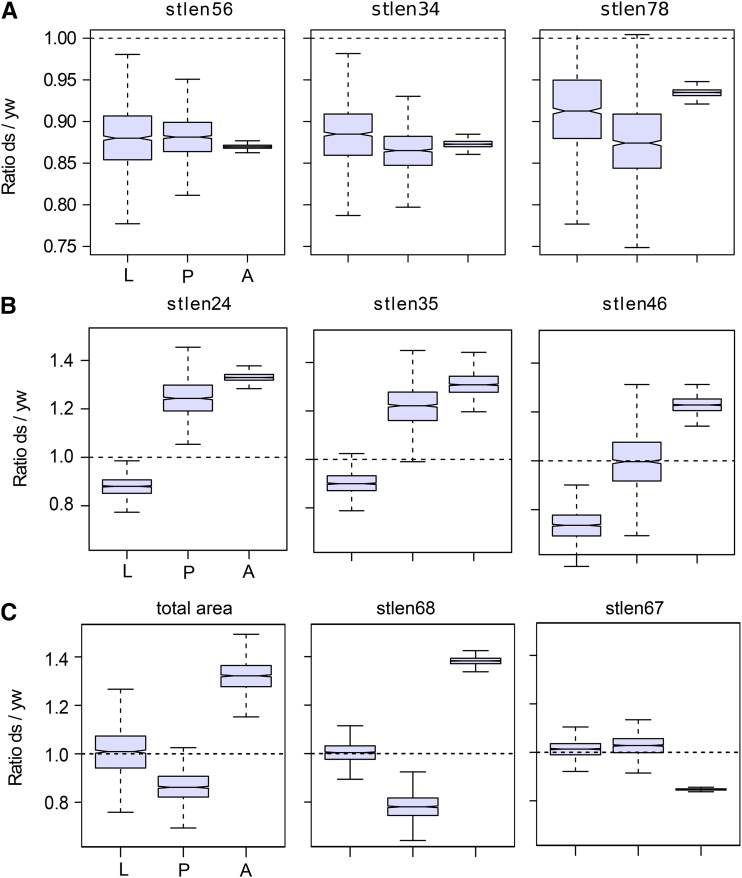
Developmental stage at which the adult wing shape differences between *ds* and *yw* appear. The boxplots show ratios of means between *yw* and *ds* genotypes at each stage for standardized distances between the pairs of landmarks (stlen) and total wing area (total area). Variance for the ratios were obtained by bootstrap (n = 1000, see methods). Notches on the boxplots display the 95% confidence interval around the median. For clarity, only few representative variables are shown (see Figure S4 for the other variables). a. Variables for which the differences between *ds* and *yw* adult wings appear before the 3^rd^ instar larval stage. b. Variables for which the differences between *ds* and *yw* adult wings appear during the larva to pupa transition. Note that for stlen46, there is a continuous increase of the ratio during larval and pupal development to reach the adult ratio. c. Variables for which the differences between *ds* and *yw* adult wings appear during the pupa to adult transition. L, larva; P, pupa; A, adult.

Examining these ratios in the larval and pupal wings shows that the differences observed between *ds* and *yw* adult wings appear at different times during development. The proximo-distal narrowing of the distal part of the wing is observed in the larval stage (Figure 8A, Figure S4A), whereas the proximo-distal lengthening of the proximal wing, as well as the broadening in the A/P axis appears at the pupal stage (Figure 8B, Figure S4B). The variation in wing area occurs mostly during the pupal to adult transition, as well as the shift of landmarks 4-6 toward the distal parts of the wing (Figure 8C, Figure S4C).

**Figure 9:**
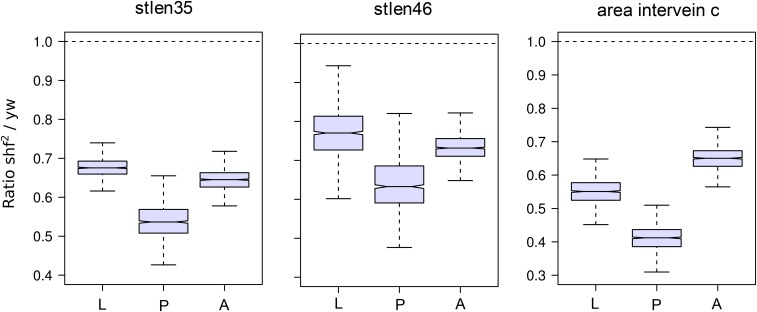
Developmental stage at which the adult wing shape differences between *shf^2^* and *yw* appear. The boxplots were obtained as in Figure 8. All major differences between *shf^2^* and *yw* adult wings are observed since the 3^rd^ instar larval stage. L, larva; P, pupa; A, adult.

Figure S5A shows the ratios *shf^2^*/*yw* for the standardized lengths in adult wings. The principal differences are the reduction of the distances between the pairs of landmarks 3-5 and 4-6, in *shf^2^*, and the corresponding reduction in that area of the wing (intervein L3L4) (Figure S5B). In the case of *shf^2^*, the differences observed in the adult wings are established in the larval wing (Figure 9), with relatively small changes after that stage.

## Discussion

Many evo-devo studies, mostly on vertebrates, succeed in linking mutations and changes in molecular signaling to quantitative natural variation in organ shape (reviewed in Parsons & Albertson 2013), but rarely provide insights into the cellular bases of the variation (*e.g.*, Nijhout *et al.*, 2014; Salazar-Ciudad & Jernvall 2010; Young *et al.*, 2010). In this context, the Drosophila wing model can help us move toward identification of the mechanistic causes of natural organ shape variation, by exposing the relative contributions of cell proliferation, cell death, cell shape, cell size, oriented division or cell polarity to organ shape variation. In Drosophila, cutting-edge tools allow live imaging developing tissues, and quantitative measurement of the dynamics of cellular processes (Etournay *et al.*, 2015; Guirao *et al.*, 2015). Such tools can readily be used to study the cellular bases of natural wing shape variation. However, in order to use these tools at the right time during development, it is important to identify the developmental stage at which the morphological variation appears. Thus, following the rationale of studies characterizing organ shape variation during ontogeny in vertebrates (*e.g.*, Martínez-Abadias *et al.* 2016; Mayer *et al.*, 2014; Young *et al.*, 2010; Zelditch & Carmichael 1989)⁠ and in Lepidoptera (Nijhout *et al.*, 2014), we have used a framework based on geometric morphometrics that allows us to quantify wing shape and size variation during development. By narrowing the time frame when differences arise, we can reduce the number of candidate developmental processes that potentially cause genotypic differences.

We tested this approach by studying wing shape variation during development in three genotypes (*yw*, *shf^2^* and *ds*) that differ in their adult wing shape, and for which there is *a priori* knowledge regarding the developmental causes of the variation, as well as regarding the developmental time during which the variation should be apparent. Our findings are consistent with this prior knowledge, suggesting that our approach can be used to identify the relevant developmental stage in cases where we have no *a priori* knowledge about the mechanisms involved in wing shape variation.

In the language of geometric morphometrics, features sharing identity or homology among specimens are referred to as landmarks. We used the relative positions of landmarks to compare changes in size and shape between developmental stages and genotypes. Both genotype to genotype and stage to stage homology assumptions could be distorted by variation in developmental timing between genotypes. The fact that we found genotypic differences consistent with *a priori* knowledge on the developmental effects of the studied alleles (see below) indicates that such artifacts, if present, did not strongly impact our analyses. However, if applied to cases where the causes of the developmental variation is unknown, special care must be taken to stage the individuals carefully to avoid this problem.

Landmark homology is not always clear when comparing specimens at different developmental stages. These uncertainties urge some caution in interpreting our results. For example, the dorsal-ventral (D-V) boundary in the larval disc is undoubtedly homologous with the wing margin in pupae and adults. On the other hand, the position of landmarks along the D-V boundary defined by Delta expression (landmarks 1, 3, 5 and 7) may be shifted along that boundary relative to those visible in the adult wing. The homology of the other four landmarks (2, 4, 6 and 8) is less assured across developmental stages, particularly when compared to the adult wing. However, it seems likely that discrepancies in the placement of these landmarks will be consistent among genotypes. If this assumption is met, differences among genotypes in how these landmarks are displaced from one developmental stage to another reflect developmental differences.

Our results could be improved through the use of more sources of data on the locations of proveins and compartment boundaries early in development. For example, staining of the Wingless expression domain in the larval and pupal wings would allow adding new landmarks by visualization of the hinge/blade boundary in the larval and pupal wings, as well as the anterior and posterior proximal margins (Kolzer *et al.*, 2003). In addition, staining L2 vein domain with antibodies against p-Mad or Srf (Cordero *et al.*, 2007) would also add a new landmark and improve wing shape measurement.

Table 4 shows that our analysis did not detect shape variation due to sex differences at any stage, although our statistical power may be low due to low sample size, and stage-to-stage variation in the the sex ratio of the samples (Table 1). Such variation in sex ratio between stages and genotypes could, in principle, affect our genotype to genotype comparisons. However, since our data are consistent with existing knowledge on the effect of the studied alleles on wing shape, such potential bias does not seem to have a strong impact on our results.

The developmental stage at which differences between the control (*yw*) and the two mutant genotype (*ds* and *shf^2^*) varied. In the case of *shf^2^*, the major pattern of variation between the adult *shf^2^* wings and the *yw* adult wings was evident at the earliest stage studied. Larval, pupal and adult *shf^2^* wings all had reduced spacing between veins L3-L4 and reduced area compared to *yw*. This suggests that the developmental processes causing this pattern of variation act early in larval development. Our findings are consistent with our *a priori* expectations based on previous studies (Glise *et al.*, 2005; Gorfinkiel *et al.*, 2005), showing that the effects of *shf^2^* on morphogenesis occur during larval development by affecting vein patterning and tissue proliferation.

In contrast, we found that the size and shape difference between *ds* and *yw* have a more complex developmental trajectory. Changes at all the developmental stages we studied contribute to the overall pattern of adult wing shape and size variation between *ds* and *yw*. Some shape differences between *ds* and *yw* appear early during larval development, others during the larval to pupal eversion, and still others during pupal development. For size, the differences appeared during pupal development. As in the case of *shf^2^*, these findings are consistent with known roles of Dachsous in epithelial morphogenesis, but they also reveal some unexpected effects. Dachsous plays an important contribution in orienting tissue growth in the direction perpendicular to the D-V boundary during larval development (Baena-López *et al.*, 2005; Mao *et al.*, 2011). Consistent with this, our data show that *ds* larval wings are slightly shorter along this axis than the wild-type. Our data are also consistent with the known effects of *ds* on pupal development (Bosveld *et al.*, 2012; Sagner *et al.*, 2012). Interestingly, the fact that additional differences become apparent during the larval to pupal transition suggest an as-yet-unidentified role for Dachsous during this transition, as well as for tissue growth during pupal development.

Our work allows us to investigate both the magnitudes of differences in shape and size, and the direction of these changes between the developmental stages studied. Consistent with the visually apparent differences in shapes among stages (*e.g.*, Figure 3), and the relatively dramatic folding and eversion that takes place during pupariation, larval wing shape differs more dramatically from pupal shape than does pupal shape from adult shape. Differences among individuals with the same genotype at the same developmental stage are noticeably smaller than those differences among genotypes. While the differences among stages and genotypes are clear, it is nevertheless apparent that the transformation that each shape undergoes during development is rather similar. This is confirmed by the relatively small angles between developmental trajectories of different genotypes.

## Conclusion

Our approach successfully identified the developmental stage at which variation appears in two cases for which the developmental causes of the variation were known. This suggests that morphometric studies of wing shape transformations in genotypes with an unknown developmental basis could provide useful hypotheses about the developmental events involved. Our approach may have particular promise when applied to natural variation in wing development, where we usually lack candidate genes to structure further investigations.
